# Neural substrates of knowledge integration: differential effects of high-definition transcranial direct current stimulation (HD-tDCS) on angular gyrus and ventrolateral prefrontal cortex

**DOI:** 10.7717/peerj.21761

**Published:** 2026-10-06

**Authors:** Xuan Zhang, Michael Lochner, Karen Joseph, Asaf Gilboa

**Affiliations:** 1 Rotman Research Institute, Baycrest Hospital, Toronto, ON, Canada; 2 Department of Psychology, University of Toronto, Toronto, ON, Canada; 3 Department of Psychology, York University, Toronto, ON, Canada; 4 Holland Bloorview Kids Rehabilitation Hospital, Toronto, ON, Canada; 5 University Health Network, Toronto, ON, Canada

**Keywords:** Semantic integration, Angular gyrus, Ventrolateral prefrontal cortex, HD-tDCS, Knowledge derivation

## Abstract

**Background:**

Humans develop extensive knowledge systems throughout their lives, which are continuously updated in response to new information. One way in which prior knowledge is updated is through semantic integration, where related semantic facts are integrated to support derivation of new information.

**Methods:**

This study investigates how anodal high-definition transcranial direct current stimulation (HD-tDCS) applied to the left angular gyrus (AG; Experiment 1) or to the left ventrolateral prefrontal cortex (vlPFC; Experiment 2) influences semantic integration and the derivation of novel information. In both studies, participants were first randomly assigned to a stimulation or sham group. Following the stimulation protocol, they were presented with a series of intermixed pairs of related facts and asked to rate their familiarity with each. Participants then completed a semantic integration test following a brief delay, answering questions about novel information that could be derived by integrating the related facts. Participants’ integration scores (primary outcome) and their correlations with general intelligence (IQ) (secondary outcome) were measured. Building on exploratory findings from Experiment 1, Experiment 2 was pre-registered to test the impact of stimulation on the correlation between IQ and integration.

**Results:**

There was no overall mean difference in integration scores between stimulation groups and their sham stimulation counterparts (all *p*s > 0.10). Stimulation to the left AG resulted in significantly lower variance in integration scores compared to sham stimulation (*p* < 0.05), reflecting fewer stimulation participants scoring very low on integration. Conversely, stimulation to the left vlPFC reduced the correlation between participants’ IQ and their integration scores (*p* < 0.05).

**Conclusion:**

Self-derivation of new knowledge is a complex, multifactorial process that was not uniquely enhanced by stimulation of the left AG or vlPFC. However, these regions appear to differentially modulate performance by reducing variability for lower-performing individuals (AG) or decoupling the dependence of performance on baseline cognitive capacity (vlPFC).

## Introduction

Humans develop large-scale knowledge systems throughout their lives (Craik & Bialystok, 2006; Piaget, 1952). These knowledge systems are continuously tuned to monitor and interpret the ongoing events and guide people’s actions, and they play a crucial role in affecting how people encode, retain, and retrieve new information (Alba & Hasher, 1983; Hintzman, 1986; Gilboa & Marlatte, 2017; Fernández & Morris, 2018; Wing et al., 2022; Wing, Gilboa & Ryan, 2024). When existing knowledge is relevant to, or overlaps with new information, prior knowledge facilitates learning (Alba & Hasher, 1983; Gilboa & Marlatte, 2017; Thorndyke & Yekovich, 1980; Wing et al., 2022; Wing, Gilboa & Ryan, 2024). Such facilitation can extend beyond direct learning, enabling the inference of new information through facilitated knowledge integration (Bauer et al., 2016; Bauer, Esposito & Daly, 2020; Bauer & Varga, 2017).

Integration-based learning engages different neural systems depending on the types of learning materials involved. Memory for arbitrary novel associations (*e.g*., learning of AB and BC pairs and inferring the AC relation) relies heavily on the active communication between the hippocampus and medial prefrontal cortex. Its structural reorganization typically requires days to fully consolidate (Guo & Yang, 2023; Preston & Eichenbaum, 2013; Schapiro et al., 2017; Schlichting, Zeithamova & Preston, 2014; Zeithamova, Schlichting & Preston, 2012). In contrast, when information is schematic or meaningful, additional cortical regions are recruited. This prior-knowledge-based learning facilitates a much faster consolidation process, which is driven by stronger cortical involvement and an earlier reduction in hippocampal dependence (Bein, Reggev & Maril, 2020; Gilboa & Marlatte, 2017; Hebscher et al., 2019; Tse et al., 2007; van Kesteren et al., 2012; Wang, Tse & Morris, 2012).

Multiple cortical regions contribute to this rapid consolidation process. In particular, the angular gyrus (AG), located in the inferior lateral parietal lobe (Binder et al., 2009; Binder & Desai, 2011; Caspers et al., 2008; Humphreys, Lambon Ralph & Simons, 2021), plays a key role in representing multimodal information (Binkofski, Klann & Caspers, 2016; Bonnici et al., 2016; Kuhnke et al., 2022; Levy, 2012; Seghier, 2012). Building on its multimodal capacity, converging evidence suggests that the AG facilitates the binding of information during semantic integration (Humphreys, Lambon Ralph & Simons, 2021; cf. Binder et al., 2009 and Binder & Desai, 2011 for other ideas that also highlight its multimodal nature). For instance, stimulating the left AG using anodal High-Definition transcranial Direct Current Stimulation (HD-tDCS) reduced participants’ reaction times when judging the relatedness of meaningful word pairs (*e.g*., plaid-jacket), compared to non-meaningful ones (*e.g*., fast-blueberry), establishing a modulatory role of the left AG in facilitating semantic associations (Price et al., 2016). Additionally, neuroimaging evidence supported this notion by demonstrating that AG activity increased in accordance with a disengagement of the hippocampus during encoding schema-congruent items (van der Linden et al., 2017). Taken together, these findings suggest that the AG’s capacity to bind multimodal information actively contributes to semantic integration. By serving as a key node for this integration, the AG facilitates a more rapid consolidation process, supporting earlier hippocampal independence (Gilboa & Marlatte, 2017; Hebscher et al., 2019).

Beyond integration, inferential processes also play a crucial role in associative learning. Whereas integration links overlapping episodes, learning by inference involves forming novel associations by selectively retrieving relevant information from prior knowledge and evaluating its applicability to the current context. This process enables connections that extend beyond direct experience and can be generalized to guide future understanding and behaviour (Becker & Cabeza, 2025; Gilboa et al., 2014, 2019; Heckers et al., 2004; Marlatte et al., 2024; Schlichting, Zeithamova & Preston, 2014; Schlichting & Preston, 2015; Zeithamova, Schlichting & Preston, 2012).

In particular, inference making requires the ability to selectively focus on relevant information while suppressing competing, irrelevant details—a capacity commonly referred to as semantic control (Badre & Wagner, 2007; Bauer & Varga, 2017; Lambon Ralph et al., 2016; Miller-Goldwater et al., 2021). Neuroimaging and lesion studies indicate that semantic control is supported by a distributed frontal and temporoparietal network, with converging evidence highlighting the critical role for the ventrolateral prefrontal cortex (vlPFC) (Binder & Desai, 2011; Lambon Ralph et al., 2016; Trambaiolli et al., 2022). The vlPFC supports semantic association and integration, particularly when task demands require selection among competing or weakly constrained meanings (Menenti et al., 2009; Sommer, 2017). Consistent with this account, lesions to the vlPFC selectively impaired the interpretation of abstract words, which place greater demands on executive regulation than concrete words (Hoffman, Jefferies & Lambon Ralph, 2010). This finding suggests that when meanings are variable or ambiguous, the vlPFC guides the selection of contextually relevant interpretations, resolving competition among alternatives and thereby supporting controlled inference derivation.

Prior study showed that successful inference derivation depends on individuals’ ability to organize relevant information. A study conducted by Varga & Manns (2021) demonstrated that individual variability in a self-derivation *via* integration task was linked to differences in participants’ cognitive capacity and neural dynamics, noting that better learning performance correlated with greater central–parietal cortical alpha synchrony. Consistent with this, the ability to integrate and self-derive novel information is robustly associated with general intelligence (IQ) and academic achievement across development (Bauer et al., 2016; Bauer, Esposito & Daly, 2020). This efficiency in deriving novel inferences is governed by underlying cognitive factors, including working memory, processing speed, abstraction, and the capacity for flexible schema creation (Benedek et al., 2017; Luchini et al., 2024).

While previous studies have implicated the AG and vlPFC in semantic integration and inference-making, their specific modulatory roles remain less understood. Furthermore, successful inference derivation exhibits significant individual variability linked to cognitive capacity (Varga & Manns, 2021), and this ability is closely tied to general intelligence (Mackintosh, 2011). Yet, how baseline intelligence interacts with the neural dynamics of integration still remains an open question. To address these gaps, the current study had two main objectives. The primary goal of this study was to examine how temporary modulation of the AG or vlPFC affects rapid knowledge integration using the self-derivation through integration task, a paradigm that has been extensively used to study semantic integration and knowledge building (Bauer & Varga, 2017; Bauer, Esposito & Daly, 2020; Esposito & Bauer, 2017, 2019; Miller-Goldwater et al., 2021; Wilson & Bauer, 2021; Varga, Esposito & Bauer, 2019). Building on this framework, the *secondary* aim of this study was to assess the relationship between IQ and knowledge integration performance, and its interaction with stimulation effects.

## Materials and Methods

To first understand the contribution of AG and vlPFC, we applied anodal HD-tDCS to either the left AG (Experiment 1) or left vlPFC (Experiment 2) before participants engaged in the learning task. Anodal stimulation was expected to increase regional cortical excitability, thereby facilitating the cognitive processes supported by the targeted area (Nitsche & Paulus, 2000; Price et al., 2016). We tested this using a self-derivation through integration paradigm, in which participants learn several intermixed pairs of trivia facts and must later integrate them to infer a novel fact. For instance, if a participant learns that “Capybara is the world’s largest rodent”, and a few items later they learn that “The world’s largest rodent lives in Savannas”, they can combine the two related yet interleaved facts to infer that “Capybara lives in Savannas”. We predicted that stimulating either the left AG or the left vlPFC would both enhance performance compared to sham stimulation. Furthermore, building on prior work linking intellectual capacity to integration performance, we predicted that anodal stimulation would impact the correlation between IQ and knowledge integration.

### Hypotheses

#### Experiment 1

Based on previous findings, we hypothesized that:

(i) HD-tDCS to the left AG would enhance participants’ integration scores compared to sham stimulation.
(ii) HD-tDCS to the left AG would lead to faster reaction times during premise information presentation.
(iii) There would be an overall positive relationship between estimated IQ and integration scores, regardless of stimulation condition.
(iv) HD-tDCS to the left AG would modulate the correlation between participants’ IQ and their integration ability.

#### Experiment 2

Experiment 2 was preregistered prior to data collection (Zhang et al., 2023, https://osf.io/dzyuw/), building on findings from Experiment 1. Because stimulation of the left AG produced largely null results, with only reduced variability among low performers, we hypothesized that targeting a region involved in semantic control (*i.e*., the left vlPFC) would yield the following performance patterns:

(i) Stimulation of left vlPFC would lead to better integration scores compared to sham stimulation.
(ii) HD-tDCS to the left vlPFC would lead to faster reaction times during premise information presentation.
(iii) There would be an overall positive relationship between estimated IQ and integration scores, regardless of stimulation condition.
(iv) Relatedly, stimulation would impact that relations such that the correlation between estimated IQ and integration score would be greater for the sham condition than the stimulation condition.
(v) There would be an interaction between the effect of stimulation and estimated IQ such that stimulation effects on integration would be particularly notable among low performers.
(vi) The effect of stimulation would be modulated by participants’ awareness of the purpose of the experiment, such that participants who were not aware of the experimental structure might show greater benefit from stimulation than those who were consciously making the inferences during the encoding phase.

### Participants

#### Experiment 1

We recruited a total of 61 participants from the Baycrest Research Participant Database at the Rotman Research Institute at Baycrest Hospital (*n*_females_ = 37). Age ranged from 19 to 35 years (*M* = 25.26, *SD* = 4.15), with an average of 16.65 years of education (*SD* = 2.47). All participants were right-handed and were proficient English speakers (*i.e*., were native English speakers or started to speak English before 12 years old and English was the dominant language they used). Participants had no history of neurological disorders (*e.g*., seizures, concussions, or other brain injuries), or psychiatric disorders. Participants were assigned alternately to either stimulation or sham group.

Data from six participants were excluded from general analyses based on pre-determined criteria specified in our preregistration (Zhang et al., 2023, https://osf.io/dzyuw/). Specifically, participants were excluded if they (a) were not fluent in English (*i.e*., began speaking or became dominant in English after age 12) (*n* = 3), (b) scored more than 2 SDs from the normative mean on intelligence measures (IQ < 70 or > 130) (*n* = 2), (c) were inattentive to the task (*e.g*., using a phone during encoding) (*n* = 1). Following the initial exclusion, we evaluated the behavioral data to ensure valid task execution. Two participants exhibited anomalous average encoding times that exceeded the group mean by more than 2 SDs; because these extreme durations suggest a failure to execute the encoding task as instructed, these cases were removed as outliers. Our final dataset comprised 53 participants (*n*_sham_ = 28, *n*_stimulation_ = 25). Additional outlier exclusion was applied to specific data analyses, which will be explained in the later sections. Participants’ consents were obtained at the beginning of the study, and they were debriefed at the end. All study procedures were approved by the Rotman Research Institute Research Ethics Board at Baycrest hospital (REB #10-61).

#### Experiment 2

Based on the results of Experiment 1, we conducted *a priori* power analysis using G*power 3.1 (Faul et al., 2009; Zhang et al., 2023, https://osf.io/dzyuw/). The analysis was specified for an F-test of a linear multiple regression model (fixed model, *R*^2^ deviation from zero), with the number of predictors set to three. Assuming a medium effect size (*f*^2^ = 0.25), a total sample size of 48 participants would be required to achieve 80% power at an α level of 0.05. A total of 52 participants was subsequently recruited for the second part of this study, from the same participant database used in Experiment 1 (*n*_females_ = 34). Participants’ age ranged from 18 to 35 years (*M* = 23.02, *SD* = 4.02) and had an average of 15.65 years of education (*SD* = 2.13). One participant was excluded from the overall analysis because despite no indication of cognitive deficits at recruitment, their estimated IQ on testing was 62, which is below the 2nd percentile for the test (suggestive of impaired intellectual functioning; see data exclusion criteria described above and preregistration at https://osf.io/dzyuw/). Thus, the final sample size of the second experiment comprised 51 participants (*n*_sham_ = 25, *n*_stimulation_ = 26).

### Stimulation paradigms

The current delivery was facilitated through a NeuroConn DC Stimulator, using a 4 × 1 multichannel stimulation device (Soterix Medical, Woodbridge, NJ, USA). Five Ag/AgCl sintered ring HD-electrodes (1.2 cm diameter) were placed within pre-attached, standard base HD-electrode holders (HD1; Soterix Medical Inc., Woodbridge, NJ, USA) filled with conductive gel (SignaGel, San Diego, CA, USA) and held in place with an elastic cap (EasyCap, Wörthsee, Germany) with marked 10–20 EEG electrode locations. In the stimulation group, the 2.0 mA current was constantly administered for a duration of 20 min, preceded by a 30-s ramp-up and a 30-s ramp-down period for a total of 21 min, which has been shown to be a safe and effective duration that achieves lasting stimulation effects (Kuo et al., 2013; Price et al., 2016). A 2.0 mA current intensity was chosen because it serves as a conventional safe upper limit in previous studies (Kuo et al., 2013; Price et al., 2016) and often leads to improved performance in domains outside the motor domain (Dedoncker et al., 2016; Price et al., 2016). In contrast, for the sham group, an identical 30-s ramp-up and a 30-s ramp-down interval was applied to capture the sensation of stimulation, which was strongest initially, with no stimulation applied for the rest of the time.

#### Experiment 1 stimulation location

For the AG stimulation, the montage configuration centered on the Montreal Neurological Institute (MNI) coordinates (−48, −65, 22) to optimize stimulation targeting the left AG (Brodmann area 39) (Collins et al., 1994; Seghier, 2012). The exact AG coordinates were selected as the location of maximal z value within AG denoting preferential activation related to the term ‘semantic’ (*p*_fdr_ < 0.01) based on a publicly available meta-analysis of 1,031 neuroimaging studies (Yarkoni et al., 2011; Neurosynth, n.d.; Fig. 1A). The anode electrode was strategically positioned at the CP5 site following the 10–20 EEG coordinate system, while the surrounding cathode electrodes were positioned at C3, P3, C7, and T7 (Fig. 1B). This configuration design allowed focal current delivery to the target anodal region, while having cathodal currents equally distributed across the four surrounding electrodes (Price et al., 2016). This paradigm was estimated to optimally stimulate the target region based on cortical electric fields computational models implemented in the SimNIBS software (Saturnino et al., 2019; Thielscher, Antunes & Saturnino, 2015) (Fig. 1C).

**Figure 1:**
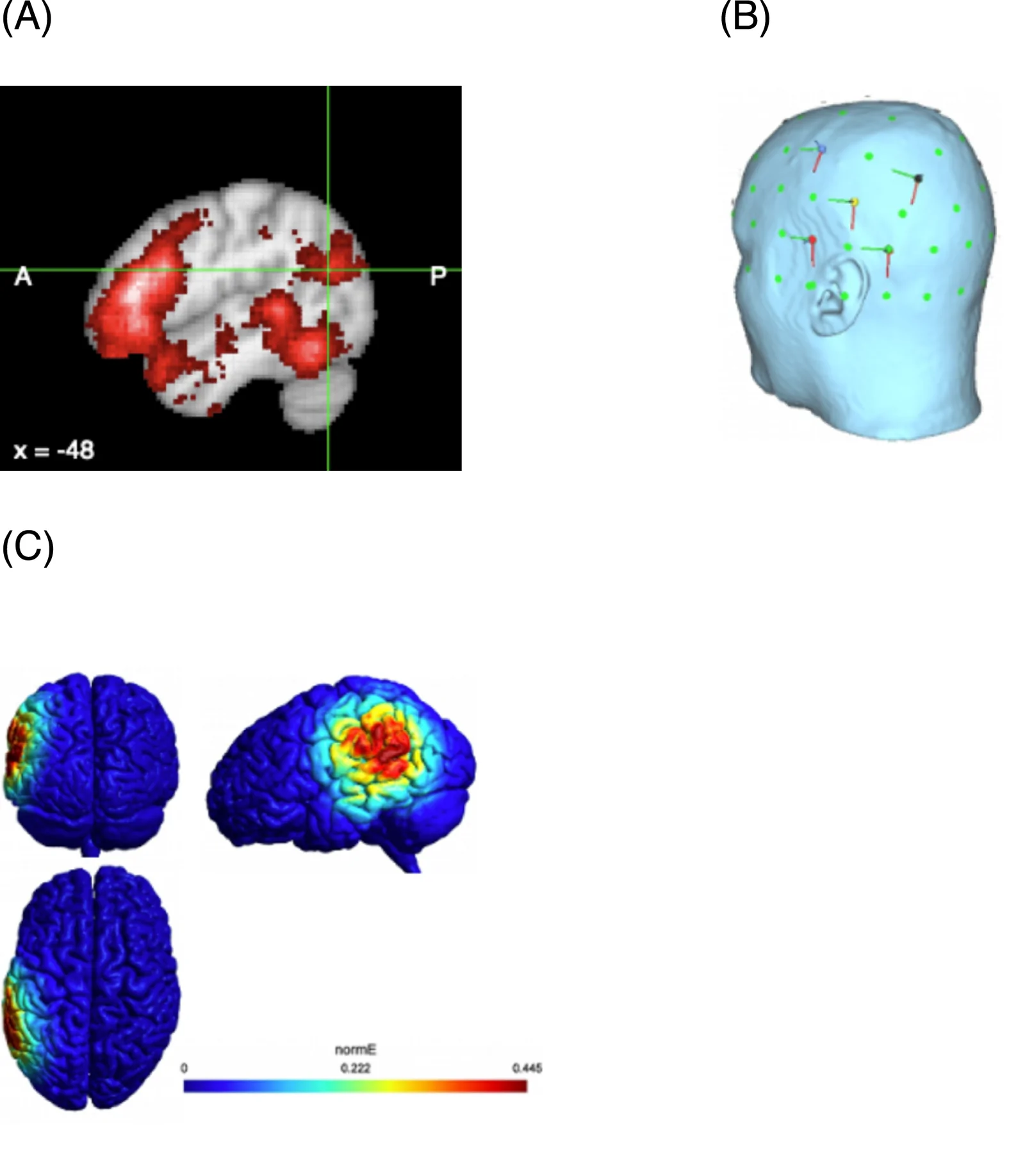
AG stimulation site. (A) Brain map showing the maximal z-score activation for the term “semantic” generated using the publicly available meta-analytic search on https://neurosynth.org, highlighting part of the left AG (x = −48, y = −65, z = 22). (B) SimNIBS-generated head model illustrating the electrode montage optimized for left AG, with the anode targeting CP5, and the surrounding cathodes targeting C3, P3, C7, T7. (C) SimNIBS-generated computational model of the cortical electric field distribution resulting in the highest stimulation strength, shown on the inflated cortical surface (at left AG). Warmer colors indicate stronger electric field intensities.

#### Experiment 2 stimulation location

Cortical electric fields computational models were implemented in the SimNIBS software to optimize stimulation effectiveness to the left vlPFC (Thielscher, Antunes & Saturnino, 2015). The model calculation output the maximum current delivered to MNI coordinates (−48, 26, −4), targeting the left inferior frontal gyrus. This resulted in an anode being situated at the F5 position, accompanied by surrounding cathodes positioned at F3, FC5, AF3, and F7, in accordance with the 10–20 EEG coordinate system (shown in Fig. 2B). This specific montage arrangement was designed to align with the MNI coordinates output by the model. The target region encompasses pars orbitalis and pars triangularis within Brodmann areas 47 and 45, which are integral regions of the vlPFC contributing to semantic cognition (Badre & Wagner, 2007; Webb, 2017). The exact coordinates were the location of maximal z value within Brodmann areas 47/45 denoting preferential activation related to the term ‘semantic’ (*p*_fdr_ < 0.01) based on a meta-analysis of 1,031 neuroimaging studies (Yarkoni et al., 2011; Neurosynth, n.d.; Fig. 2A). The rest of stimulation paradigms remain consistent with those used in Experiment 1.

**Figure 2:**
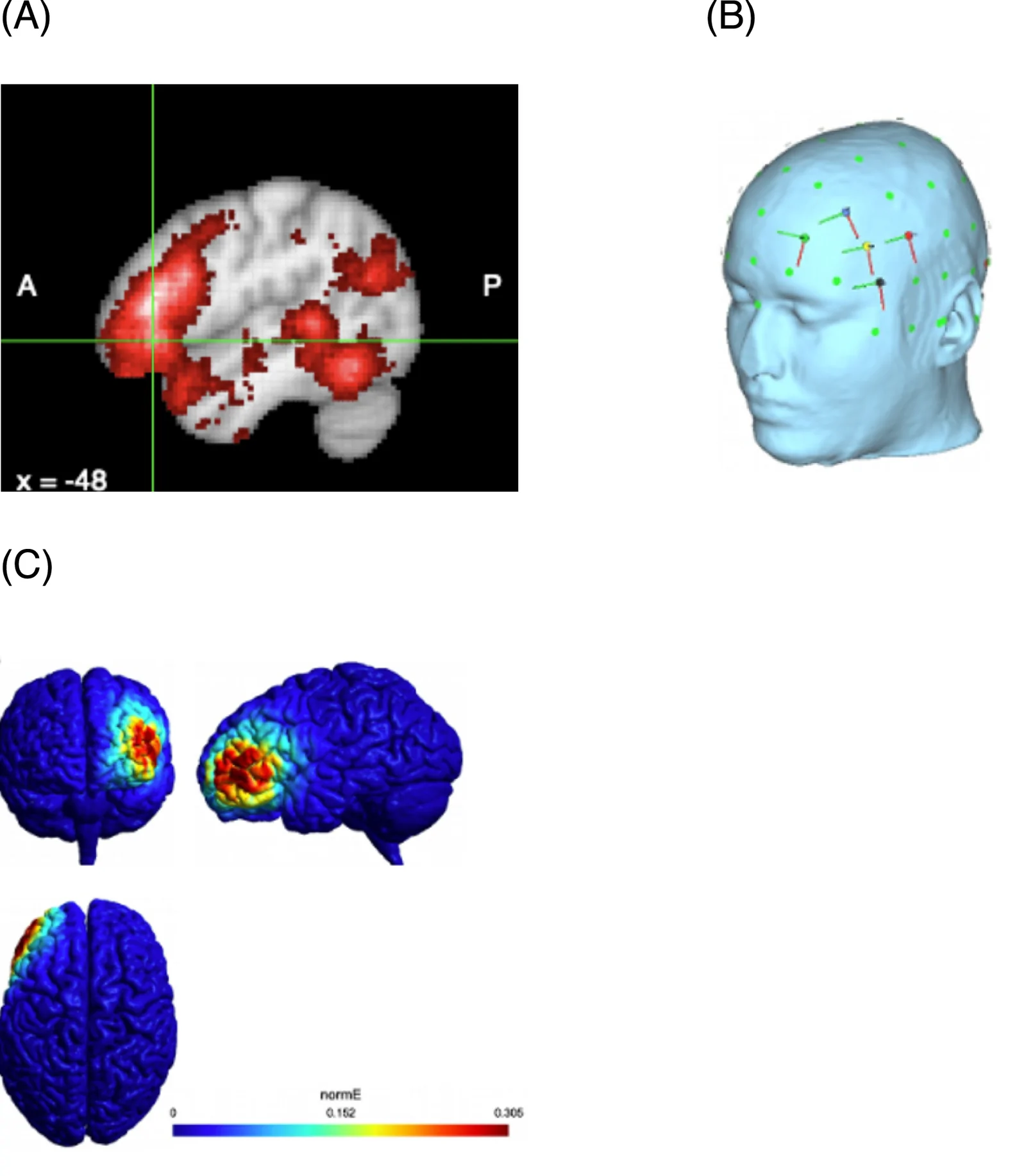
vlPFC stimulation site. (A) Brain map showing the z-score activation for the term “semantic” generated using the publicly available meta-analytic search on https://neurosynth.org, highlighting part of the left inferior frontal gyrus (x = −48, 26, −4). (B) SimNIBS-generated head model illustrating the electrode montage optimized for left vlPFC, with the anode targeting F5, and the surrounding cathodes targeting F3, FC5, AF3, and F7. (C) SimNIBS-generated computational model of the cortical electric field distribution resulting in the highest stimulation strength, shown on the inflated cortical surface (centered on the anterior pars triangularis and the pars orbitalis of the vlPFC). Warmer colors indicate stronger electric field intensities.

### Stimuli

This study adopted a self-derivation through integration task used in Bauer & Varga (2017). Thirty pairs of novel facts (*i.e*., a total of 60 facts) were selected. Each pair of facts can be integrated to allow participants to derive a novel target fact (Bauer, 2020; BLISS numbers S001-S030). For instance, two stem facts could be shown as “Cyanide is found in pips” and “Apple seeds are called pips” (Figs. 3A, 3B). The integration target fact would be “Apple seeds contain cyanide” (Fig. 3C). Facts were four to 10 words in length and covered a range of topics such as arts, history, and science. The order of facts was pseudo-randomized prior to the study with two to four intervening facts inserted between each pair. This lag was designed to ensure participants are required to retrieve the prior fact in order to complete the derivation (Bauer & Varga, 2017; Varga & Manns, 2021). Facts were presented one word at a time for 500 ms, At the end of each sentence, participants were prompted with a question “Did you know this fact before?”. This question aimed to test participants prior knowledge of each fact to ensure that they were unfamiliar with most of the facts presented. Reaction times and button responses were collected when participants clicked “yes” or “no”. Facts were presented using cognition.run (Cognition, n.d.).

**Figure 3:**
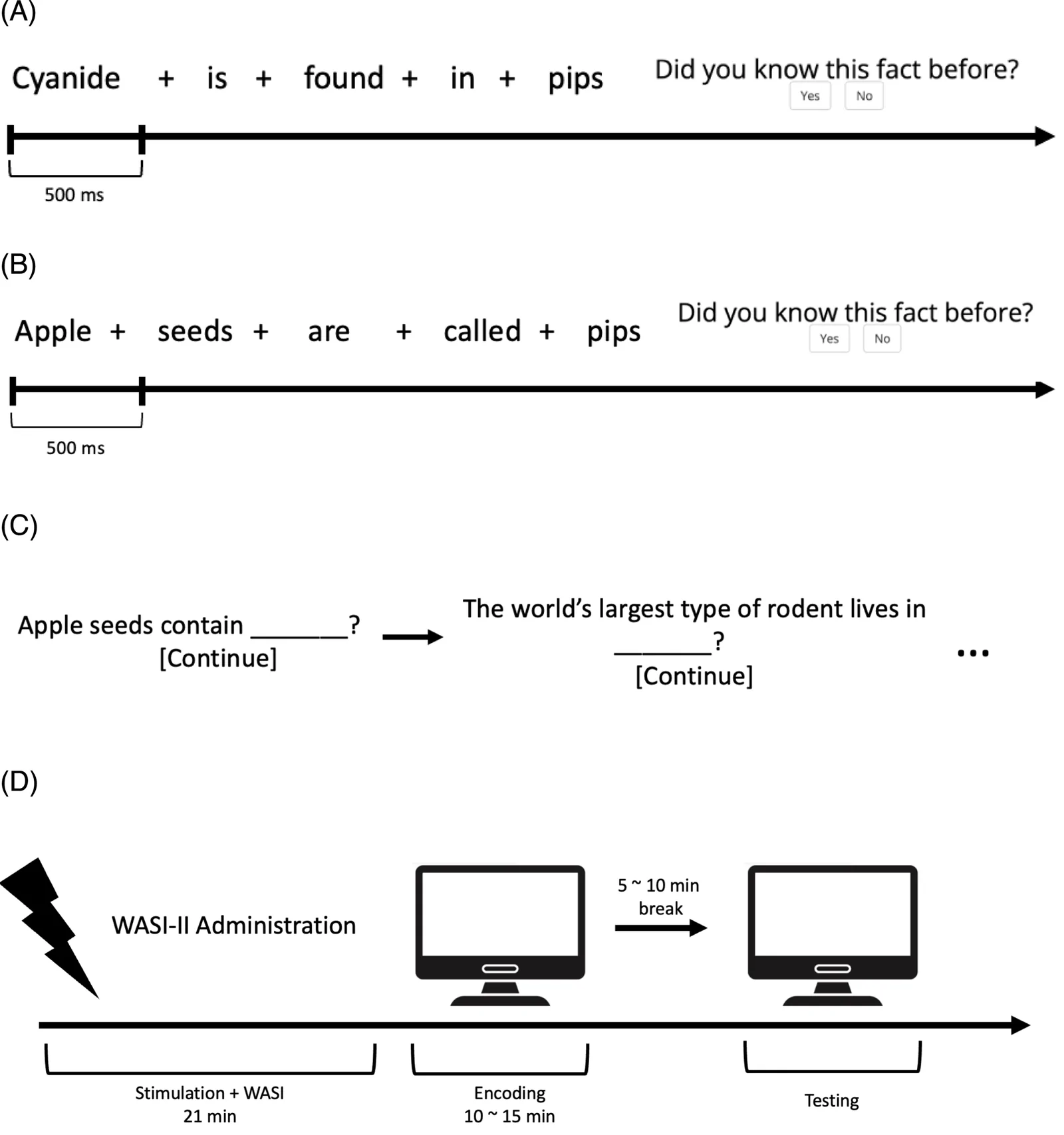
Experiment stimuli and procedure. (A) During encoding, participants viewed semantically related fact pairs, presented one word at a time, with each word shown for 500 ms. After each fact, participants indicated whether they had known it previously. (B) After two to four intervening sentences, a second fact that shared an overlapping element was presented in a similar format. (C) In the derivation task, participants were then tested on their ability to derive new information by integrating across the two presented facts. (D) An overview of the experimental paradigm.

Experiment 2 was identical, except that additional questions and Likert scale items were added to the post-stimulation questionnaire to assess participants’ awareness to the fact pairs more directly. Participants first responded to general questions about what they thought the experiment was about (*e.g*., did you notice anything odd or interesting about the facts?) followed by more specific and graded questions about the association between the fact pairs (*e.g*., on a scale of 1–5, as you were reading, how related did you think some of these facts were?) (for more details, see Supplemental Materials).

### Procedure

Written informed consent was obtained from all participants prior to participation. Head circumference was measured to determine the appropriate cap size for each participant (56, 58, or 60 cm). For fitting electrodes, electrode holders were pre-attached to the cap, and the electrodes were placed in the holders which were filled with conductive gel. The other side of the electrodes were then connected to a multichannel transcranial electrical stimulator (DC-Stimulator MC; Neuroconn, Ilmenau, Germany). Cotton swabs were used to move hair aside to ensure an impedance of less than 5k ohms was reached, measured with a portable impedance meter (Meltzer et al., 2024).

During stimulation, participants’ IQ were assessed using the WASI-II verbal and matrix reasoning subsets, and their responses were recorded manually by experimenters (Wechsler, 2011). The stimulation phase lasted 21 min for both the stimulation and sham group. After the stimulation period, participants commenced on the self-derivation through integration task. Participants were instructed to engage in a novel fact learning task in the following section, and they were also instructed to focus on the task and keep their electronic devices away while completing this task. The encoding task lasted around 10 to 15 min (Fig. 3D).

Following a brief intermission lasting 5 to 10 min, participants continued on the self-derivation task. Thirty questions were presented one at a time on the screen with the final word missing (*e.g*., The world’s largest type of rodent lives in _____?). Participants were given unlimited time to provide their answers verbally, and the experimenter recorded the answer on a testing sheet. Upon completion of one question, participants clicked on the “continue” button on the screen and moved on to the next question. In certain instances, participants might provide an answer that did not necessitate the integration of previously learned fact pairs. In such cases, participants were then prompted by the experimenter with an additional question “Do you want to give another word that may complete this sentence?” to potentially induce the integration of pertinent facts (Varga, Cronin-Golomb & Bauer, 2022). After the self-derivation test, participants were given a brain stimulation questionnaire to assess any side effects that may have resulted from the stimulation, and whether they noticed if they were on the stimulation or sham group. Finally, a questionnaire assessing awareness of the structure of the experiment and of the potential relationships between fact pairs was administered.

### Analysis

#### Experiment 1

Statistical analyses were conducted using R (version 3.6.0; R Core Team, 2019). To examine the stimulation effects on participants’ integration performance during the self-derivation task, a Levene’s test for equality of variances was conducted to compare variance in integration scores between the stimulation and sham group. If the assumption of equal variances was violated, a Welch’s t test was then applied to compare the mean difference of integration scores between the stimulation and sham group. To calculate the integration scores, participants received a score of “1” for each correct answer, including the ones from the follow-up prompts. The integration scores were then calculated as the total number of correct responses, with scores ranging from 0 to 30 (maximum).

Regarding the second hypothesis about the stimulation effect on encoding reaction time, an independent samples t-test was conducted to compare the reaction times between the stimulation and sham groups. Three participants’ reaction time data were excluded from this analysis due to being 2 SDs above the RT mean (all from the sham group). This test aimed to determine if stimulation significantly improved processing speeds (cf. Price et al., 2016).

An estimated IQ was derived from the combination of the verbal and matrix reasoning subset scores of WASI-II (Wechsler, 2011). To examine the relationship between intelligence and fact integration ability, Pearson’s correlations were computed between participants’ IQ and integration scores. A Fisher’s *Z* test was then applied to compare the strength of these correlations between the stimulation and sham groups. This analysis assessed whether higher intelligence was associated with better fact integration and whether stimulation modulated this relationship.

Finally, participants’ awareness of fact pairs was evaluated using a post-stimulation questionnaire (detailed in the Supplemental Section). A dichotomous awareness scoring system was applied, where ‘1’ indicated awareness of any association between fact pairs, and ‘0’ reflecting no awareness. Data from three participants who did not complete the questionnaire were excluded (one from the stimulation group and two from the sham group). To examine whether awareness of fact pairs influenced integration performance, an independent samples t-test compared integration scores between the aware and non-aware group.

#### Experiment 2

To evaluate the effects of vlPFC stimulation in Experiment 2, we first compared integration performance between groups using an independent-samples t-test, having confirmed homogeneity of variance *via* Levene’s test. Similarly, group differences in encoding reaction time were assessed using an independent-samples t-test; for this analysis, two participants from the stimulation group were excluded as outliers (RT > 2 SDs above the mean). We then examined the relationship between full-scale IQ and integration performance using Pearson’s correlations, with coefficients compared across conditions *via* a Fisher’s *Z* test.

Finally, to more directly evaluate the effects of awareness on knowledge integration, the awareness assessment was expanded in Experiment 2. While Experiment 1 utilized a dichotomous “notice” check, Experiment 2 included both an explicit binary probing question (*e.g*., identifying any relationships between items) and a 5-point Likert scale to capture graded individual differences in awareness intensity (1 = not related at all, 5 = very related). Similar to Experiment 1, if participants’ answers mentioned any association between fact pairs, they received an awareness score of “1” for the binary awareness question. The binary scores and Likert ratings were then fit into a linear regression to predict integration performance. To determine the most statistically robust predictor of performance, we performed a systematic model comparison using the Akaike Information Criterion (AIC; Burnham & Anderson, 2004). We compared the predictive power of the binary awareness scores against the continuous Likert ratings. The Likert-scale model provided a superior fit to the data, justifying its use as the primary awareness metric. Following this model selection, we calculated the Pearson correlation between awareness ratings and integration performance. Furthermore, we fit a linear model treating awareness as a moderator and test group as a main predictor to evaluate whether awareness would modulate stimulation effects on integration scores. Since the analysis encountered multicollinearity issues between test groups and awareness levels, awareness scores were mean-centered before running the analysis (Model 1).

#### Model 1

Integration scores ~ Awareness scores (centered) + Test groups + Awareness scores (centered) * Test groups

## Results

### Experiment 1

Participants in the sham or stimulation group were unable to guess which group they were in, as indicated by their post-stimulation questionnaire answers (questionnaire shown in Supplementary). Due to the small cell frequencies in the contingency table, a Fisher’s exact test was employed. Results showed insignificant difference between groups (*p* = 1.00, *φ* = 0.00). Participants’ responses during the encoding session indicated a lack of familiarity with most of the facts, with a mean familiarity rating of 27.45% (out of 60 facts). Participants in both groups demonstrated similar WASI scores (*M*_stim_ = 102.24, *SD*_stim_ = 7.95; *M*_sham_ = 98.75, *SD*_sham_ = 11.01), and an independent samples *t*-test showed that stimulation did not impact participants’ IQ scores, despite WASI tests being conducted within the stimulation period (*t*(51) = 1.31, *p* = 0.197, *d* = 0.36).

#### AG stimulation effects on integration performance and reaction time

A Levene’s test revealed a significant difference in integration data variances between the stimulation and sham group, *F*(1, 51) = 4.14, *p* = 0.047, with the variance (*s*^2^_stim_ = 32.29) of the stimulation group lower than the variance of the sham (*s*^2^_sham_ = 69.36) (Fig. 4A). Since the assumption of homogeneity was violated, a Welch’s t test was conducted, and it indicated no significant difference in participants’ integration scores between the stimulation (*M*_stim_ = 15.04, *SD*_stim_ = 5.68) and sham groups (*M*_sham_ = 12.39, *SD*_sham_ = 8.33), *t*(47.86) = 1.36, *p* = 0.179, Cohen’s *d* = 0.37 (small). This result suggests that HD-tDCS stimulation of the left AG did not enhance participants’ overall performance on the self-derivation task (Fig. 4A).

**Figure 4:**
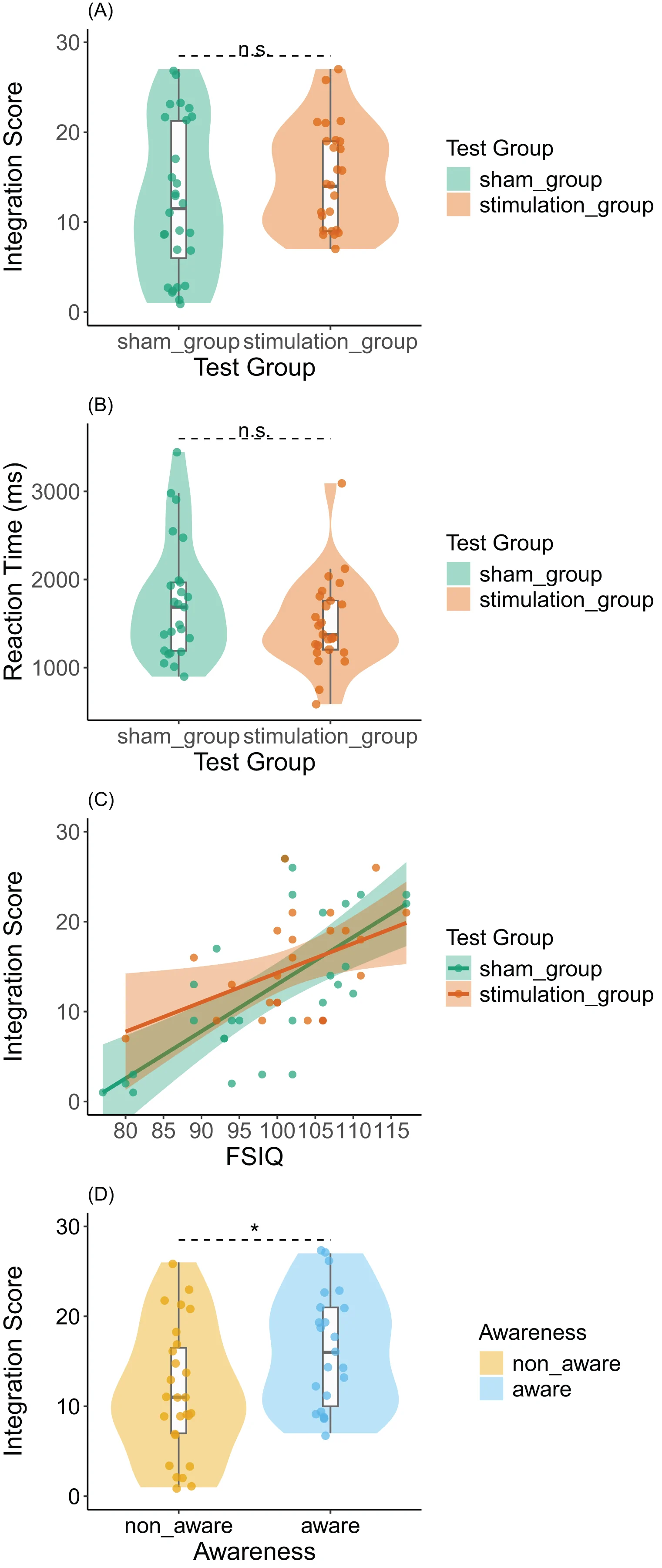
Integration performance by test groups, reaction time by test groups, and integration performance by awareness groups. (A) Self-derivation results of the stimulation and sham group. A Levene’s test revealed a significant variance difference between the sub-groups (*p* = 0.047, *S*^2^_stimulate_ = 32.29, *S*^2^_sham_ = 69.36), but a Welch’s t test did not reveal significant difference in integration scores (*p* = 0.179, *M*_stimulate_ = 15.04, *M*_sham_ = 12.39). (B) Reaction time differences between the stimulation and sham groups. Independent samples t test revealed a facilitation effect for the stimulation group (*M* = 1,500.22 ms) compared to the sham (*M* = 1,749.56 ms), though not significant (*p* = 0.143). (C) Correlation results between FSIQ and integration scores. Pearson’s correlations were significant for both test groups (*r*_stimulate_ = 0.46, *r*_sham_ = 0.69, ps ≤ 0.022 < 0.001). (D) Self-derivation results of the aware and non-aware groups. An independent samples t test revealed a significant group difference in integration scores (*M*_aware_ = 16.30, *M*_non-aware_ = 11.48, *p* = 0.017).

To examine participants’ processing speed, an independent samples *t*-test was conducted. The results revealed no significant difference in reaction times between the stimulation and sham groups, *t*(48) = 1.49, *p* = 0.143. The stimulation group showed numerically faster non-significant average reaction time (*M*_stim_ = 1,500.22, *SD*_stim_ = 504.27) compared to the sham group (*M*_sham_ = 1,749.56, *SD*_sham_ = 669.12), with a small effect size Cohen’s *d* = 0.42 (Fig. 4B). This suggests that HD-tDCS stimulation to the left AG did not significantly enhance the processing speed for accessing knowledge to decide whether facts presented are novel or not.

#### AG stimulation effects on integration performance and IQ

A Pearson’s correlation test revealed a large and significant overall positive correlation between participants’ IQ and their integration scores, *r*(51) = 0.63, *p* < 0.001. This positive correlation was found to be moderate and significant within the stimulation group (*r*(23) = 0.46, *p* = 0.022) and large and significant in the sham group (*r*(26) = 0.69, *p* < 0.001) (Fig. 4C). A Fisher’s *Z* test revealed this difference in correlation size between sub-groups was not significant (*Z* = 1.23, *p* = 0.219). It highlights that participants with higher IQ tended to integrate more facts compared to those with lower IQ, regardless of which group they were in.

#### Impact of awareness ratings on integration performance

After confirming the homogeneity of variance using Levene’s test, *F*(1, 48) = 0.07, *p* = 0.782, an independent samples *t*-test was conducted to investigate whether participants’ integration abilities were influenced by their awareness. The result indicated a significant group difference, *t*(48) = 2.47, *p* = 0.017, Cohen’s *d* = 0.70 (Fig. 4D), showing that participants who indicated awareness of the association between fact pairs tended to integrate more facts (*M*_aware_ = 16.30, *SD*_aware_ = 6.40) than participants who were not aware of the associations (*M*_non-aware_ = 11.48, *SD*_non-aware_ = 7.25).

In summary, anodal HD-tDCS of the left angular gyrus (AG) did not produce significant effects on integration scores or reaction times. However, score variance differed between groups, with the stimulation group showing reduced variability relative to sham, suggesting a potential modulation of performance among lower-performing participants. Integration scores were strongly and positively correlated with IQ, replicating prior findings, but this relationship was not significantly altered by stimulation, indicating that left AG activity may not directly underlie the association between reasoning ability and self-derivation. Participants who reported awareness of fact-pair associations demonstrated higher integration performance. Given the categorical nature of the awareness measure in Experiment 1, Experiment 2 incorporated Likert-scale ratings to enhance sensitivity and validity.

### Experiment 2

Because stimulation of the left AG produced largely null main effects with only reduced variability among low performers, but exploratory analyses revealed differential effects of IQ and awareness on performance, these effects were explicitly included as secondary hypotheses in the preregistration of Experiment 2. As in Experiment 1, participants were unable to distinguish whether they were in the stimulation or sham group (*p* = 1.00, *φ* = 0.00). Their responses during the encoding session indicated a lack of familiarity with most of the facts, reflected by a mean familiarity rating of 20.65% (out of 60 facts). Both groups had similar WASI scores (*M*_stim_ = 99.88, *SD*_stim_ = 8.91; *M*_sham_ = 96.88, *SD*_sham_ = 8.56), and an independent samples *t*-test showed that the stimulation did not affect participants’ performance on IQ test (*t*(49) = 1.23, *p* = 0.226, *d* = 0.34).

#### vlPFC stimulation effects on integration performance and reaction time

A Levene’s test revealed non-significant variance difference between the stimulation and sham group, *F*(1, 49) = 2.50, *p* = 0.121, confirming the homogeneity of data variances. An independent samples *t*-test was subsequently conducted, revealing a non-significant integration score difference between the stimulation (*M*_stim_ = 15.62, *SD*_stim_ = 7.60) and the sham group (*M*_sham_ = 14.40, *SD*_sham_ = 6.34), *t*(49) = 0.54, *p* = 0.539, Cohen’s *d* = 0.17 (Fig. 5A). This result suggests that contrary to our hypothesis, HD-tDCS to the left vlPFC did not significantly enhance participants’ ability to integrate fact pairs.

**Figure 5:**
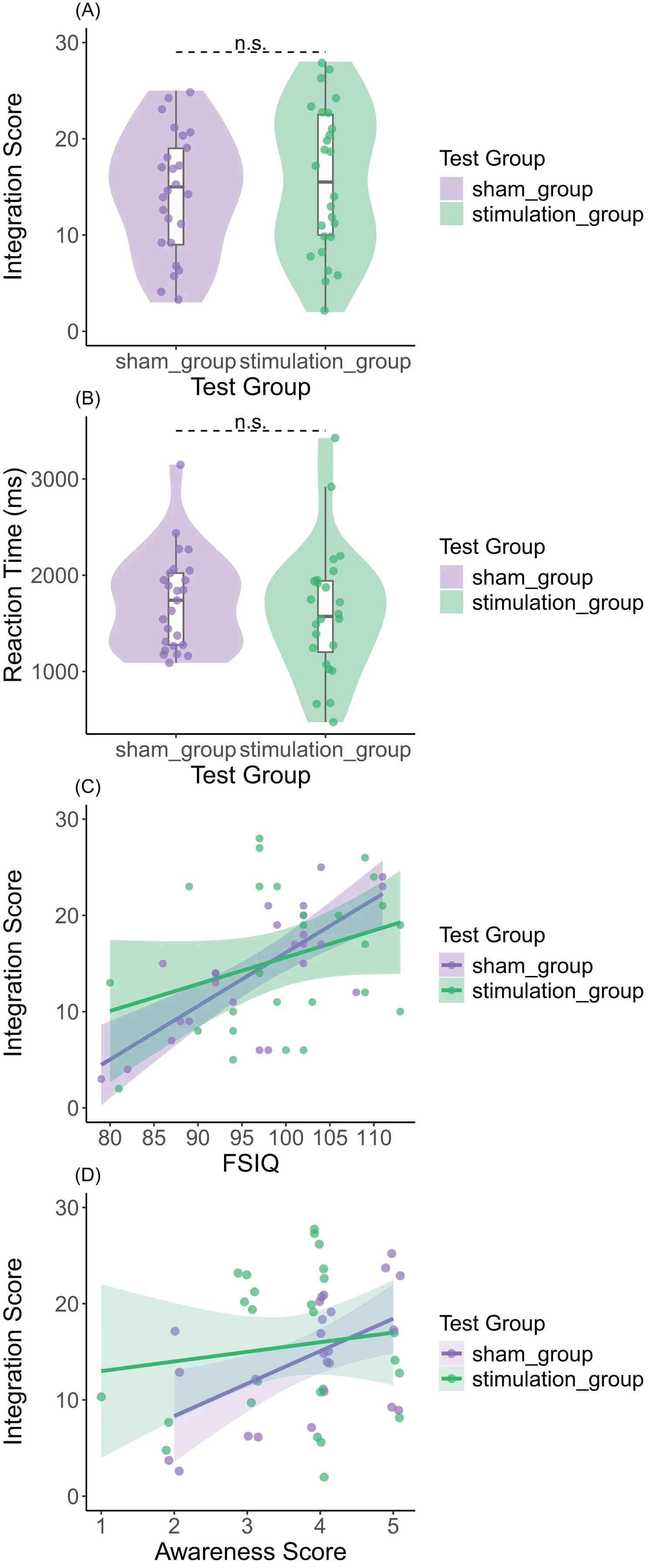
Integration performance by test group, reaction time by test group, association between IQ and integration scores, association between awareness ratings and integration performance. (A) Self-derivation results of the stimulation and sham group. An independent samples t test did not reveal significant difference in integration scores (*M*_stimulation_ = 15.62, *M*_sham_ = 14.40, *p* = 0.539). (B) Reaction time difference between the stimulation and sham group. An independent samples t test did not reveal facilitation effect (*p* = 0.542) for the stimulation group (*M*_stimulation_ = 1,620.61), compared to the sham (*M*_sham_ = 1,725.12). (C) Correlation results between IQ and integration scores. A Fisher’s Z test demonstrated a significant correlation difference between the stimulation and the sham (*r*_stimulate_ = 0.33, *r*_sham_ = 0.75, *p* = 0.034), such that the positive correlation for stimulation group was reduced. (D) Pearson’s correlation and moderation regression results both demonstrated a significant association between explicit awareness ratings and integration scores (*r*(49) = 0.30, *p* = 0.034; B = 3.38, SE = 1.37, t *= 2.47, p* = 0.017). However, no significant interaction was found (*p* = 0.225).

Further, an independent samples *t*-test was conducted to assess the reaction time difference between stimulation and sham group, after confirming homogeneity of variance using Levene’s test (*F*(1, 47) = 0.90, *p* = 0.349). The results revealed no significant encoding time difference between the stimulation (*M*_stim_ = 1,620.61, *SD*_stim_ = 680.41) and sham group (*M*_sham_ = 1,725.12, *SD*_sham_ = 499.89), *t*(47) = 0.61, *p* = 0.542, Cohen’s *d* = 0.21 (Fig. 5B). This suggests that HD-tDCS stimulation to the left vlPFC did not enhance participants’ reaction time during encoding.

#### vlPFC stimulation effects on integration performance and IQ

Pearson’s correlation test showed an overall moderate positive correlation between IQ and integration scores, *r*(49) = 0.51, *p* < 0.001, demonstrating that participants with higher IQ were likely to achieve higher scores in integration task replicating previous findings. Further analysis with separate Pearson’s correlation tests for the stimulation and sham groups revealed a significant difference in correlation coefficients (Fisher’s *Z* = 2.12, *p* = 0.034). Consistent with our hypothesis, participants in the stimulation group showed a non-significant moderate sized positive correlation (*r*(24) = 0.33, *p* = 0.103), whereas the sham group maintained a large and significant positive correlation (*r*(23) = 0.75, *p* < 0.001) (Fig. 5C). This suggests that stimulation attenuated the relationship between IQ and integration performance relative to the sham condition. To examine how stimulation effects may vary across IQ ranges, we performed exploratory non-parametric subgroup analyses. These results are provided in the Supplemental Materials.

#### The effects of explicit awareness ratings and fact integration

To assess the effect of participants’ awareness on integration scores, we first compared the predictive power of the binary awareness check and the Likert-scale ratings using independent linear regression models. The continuous Likert-scale ratings were a significant predictor of integration performance, *B* = 2.11, *SE* = 0.96, *t* = 2.18, *p* = 0.034, *R*^2^_*adj*_ = 0.07, whereas the binary ratings were not, *B* = 2.86, *SE* = 2.55, *t* = 1.12, *p* = 0.268, *R*^2^_*adj*_ = 0.01. Formal model comparison confirmed that the continuous model provided a superior fit to the data (*AIC* = 342.98) compared to the binary model (*AIC* = 346.43), with a relative marginal likelihood of 0.18. Based on this evidence, subsequent analyses were conducted using the continuous awareness scale.

A Pearson’s correlation revealed a significant positive relationship between awareness ratings and integration scores, *r*(49) = 0.30, *p* = 0.034. This finding replicates the result from Experiment 1, demonstrating that higher awareness of fact pairs was associated with higher integration scores. To investigate whether stimulation differentially benefited participants based on their awareness, a moderation analysis was conducted. The overall model was trending towards significance, *F*(3, 47) = 2.35, *p* = 0.085, *R*^2^_*adj*_ = 0.08. Mean-centered awareness significantly predicted integration scores, *B* = 3.38, *SE* = 1.37, *t* = 2.47, *p* = 0.017, indicating that higher awareness scores were associated with better integration performance. However, the interaction between awareness and test group was not statistically significant, *B* = −2.38, *SE* = 1.93, *t* = −1.23, *p* = 0.225 (Fig. 5D). This suggests that the relationship between awareness and integration scores did not significantly differ between the sham and stimulation conditions.

In Experiment 2, anodal HD-tDCS to the left vlPFC did not significantly enhance integration scores or facilitate reaction times, contrary to our hypotheses. This is similar to the findings from Experiment 1, where stimulation of the left AG also did not enhance integration scores or encoding reaction times. These results suggest that these two regions may not crucially contribute to self-derivation, as stimulation of these regions separately did not produce significant changes. By contrast, the analysis revealed a reduction in the correlation between IQ and integration scores in the stimulation group but not in the sham group. This indicates that stimulation to the left vlPFC significantly dampened the relationship between IQ and integration scores (Fig. 5C). Finally, participants’ explicit Likert scale ratings of awareness significantly correlated with their integration scores. This suggests that similar to Experiment 1, participants who were more aware of the associations between fact pairs were more likely to score better on the self-derivation task. However, this correlation was not influenced by the stimulation, which was confirmed by the moderation analysis. This indicates that HD-tDCS to the left vlPFC did not affect participants’ levels of awareness.

## Discussion

The AG and vlPFC are central to semantic processing. The current study examined their specific roles in semantic integration through self-derivation. Contrary to our hypotheses, stimulation of neither structure significantly enhanced participants’ mean integration scores, suggesting that at the group level these regions are unlikely to causally contribute to semantic integration as measured by the self-derivation paradigm. However, stimulation to each region produced differential effects that appeared to modulate the integration scores of participants. Stimulation to the left AG resulted in significantly smaller integration variance in the stimulation group, particularly increasing the lower boundary of the performance range. Conversely, stimulation to the left vlPFC attenuated the correlation between participants’ IQ and their integration scores. These findings suggest differential but potentially related roles for the AG and vlPFC in semantic integration *via* self-derivation.

### Anodal stimulation of the left AG or left vlPFC does not improve semantic integration

Stimulation to the left AG or the left vlPFC did not significantly enhance participants’ integration scores. Multiple factors may contribute to these null results. One possibility is that the left AG and left vlPFC may not contribute crucially to self-derivation because of the task’s multifactorial nature. Although there is considerable evidence supporting their contribution to semantic processing, the tasks used in these studies might tap into sub-processes that only contribute to concept-specific semantic integration when elements are directly provided. Therefore, enhancements observed in previous studies may not generalize to the current study due to the behavioral paradigm we employed, which requires self-derivation of overarching, multiplex combination of prior knowledge elements across time. By contrast, many of the studies that have suggested the AG’s role in combinatorial semantics and modality integration, have used in-the-moment processing of word-level stimuli and specified semantic or thematic categories (Bonner et al., 2013; Molinaro et al., 2015; Price et al., 2015, 2016). For instance, Price et al. (2016) showed that stimulation to the left AG facilitated reaction times during a semantic relatedness task, by using in-the-moment meaningful word pairs. The discrepancy between the results of previous studies and those of the present study indicates that the left AG may be involved in semantic integration when relevant elements are processed concurrently, but may not be a critical structure when disparate elements are processed across time.

The experimental task of this study loads heavily on cognitive control for off-line self-derivation of semantic information. We therefore expected that stimulation of the left vlPFC, which plays a role in cognitive control of semantics, would impact integration, despite the lack of effect of AG stimulation. However, we found no evidence that stimulation of vlPFC impacts semantic integration on average. This may be due to the multifactorial nature of self-derivation, which involves not only semantic control but also verbal comprehension, working memory, inhibitory control, memory integration and meta-cognition (Miller-Goldwater et al., 2021). This multi-determination of outcome might explain why altering the function of a single anatomical structure does not, on average, alter the outcome despite vlPFC’s role in meta-cognitive processes. In contrast, tasks that target specific semantic contrasts (*e.g*., abstract *vs*. concrete word processing) may yield more robust effects of vlPFC modulation, as abstract words typically engage greater executive control (Hoffman, Jefferies & Lambon Ralph, 2010).

In addition to task complexity, technical limitations of HD-tDCS may further constrain observable effects. Stimulation at 2.0 mA may be insufficient to reliably modulate neural processing under the high cognitive demands imposed by the self-derivation through integration task. Moreover, the highly focal nature of HD-tDCS may limit its ability to effectively engage the distributed semantic control network, particularly given inter-individual variability in the anatomical organization of semantic regions. These factors may reduce sensitivity to mean-level behavioral effects, even if stimulation alters underlying neural dynamics.

Consistent with a network-level account, Varga & Manns (2021) found strong alpha coherence between frontal and parietal regions using EEG to examine self-derivation, suggesting coordinated interactions between control- and representation-related regions. Rather than indicating a direct causal role in integration *per se*, the present findings support the modulatory involvement of the vlPFC and AG in self-derivation, shaping performance variability and its dependence on cognitive capacity within a distributed semantic network. Thus, examining network-wide cortical dynamics following stimulation may reveal changes in functional connectivity that do not necessarily translate into coarse behavioral differences, but instead reflect more subtle, process-level effects. In addition to AG and vlPFC, other regions, such as the anterior and lateral temporal cortices and midline fronto-parietal areas have also been implicated in semantic integration (Binder & Desai, 2011; Binder et al., 2009; Branzi et al., 2021; Branzi & Lambon Ralph, 2023; Merhav, Karni & Gilboa, 2015). Stimulation-induced changes may therefore emerge through altered interactions across this broader semantic network, rather than through modulation of a single node.

### Differential stimulation effects of AG and vlPFC

While stimulation to the left AG and vlPFC did not enhance participants’ overall integration performance, it produced a pattern of results suggestive of unique and differential modulation of group performance. In Experiment 1, evaluation of group variance—conducted as a pre-analysis diagnostic step—revealed that left AG stimulation significantly reduced the variance of integration scores, particularly among lower-performing participants. This exploratory observation suggests individual differences between high and low performers in the self-derivation task. This effect is consistent with findings reported by Varga & Manns (2021) who found high performers and low performers showed different brain oscillations when completing the self-derivation task. Specifically, high performers had increased oscillatory activity in the alpha band over posterior central-parietal regions, and this was modulated by slower delta wave. Several studies have shown increases in alpha frequency range following anodal stimulation of parietal cortex (Azarpaikan et al., 2019; Chen et al., 2022; Spitoni et al., 2013). It could be that low performers in our study specifically benefited from stimulation-induced alpha increases which was not beneficial for those already performing at higher levels, as high performers already have heightened alpha oscillation during knowledge integration. The present study did not record EEG, and this hypothesis remains to be validated.

Stimulation to the left vlPFC also appeared to modulate performance by altering the typical relationship between cognitive capacity and integration. Specifically, while executive function is a strong predictor of integration performance on this task (Esposito & Bauer, 2017; Varga, Esposito & Bauer, 2019), which was also found in the current study (with an IQ measure), this robust association was attenuated following the vlPFC stimulation. The mechanism underlying this decoupling remains unclear. One possibility, explored descriptively in the Supplemental Materials, is that decoupling was driven by individuals at the lower end of the IQ range performing better than expected. Alternatively, stimulation may have introduced greater variability in performance across participants, reflecting individual differences in HD-tDCS responsiveness. Individuals with higher cognitive abilities often demonstrate enhanced pattern recognition and working memory to streamline information assimilation (Jensen, 2006; Kovacs & Conway, 2016; Lee et al., 2011; Ling & Loh, 2021; Shelton et al., 2010). Given that the vlPFC lies at the intersection of attention and executive networks and supports both executive functions and semantic control (Badre et al., 2005; Badre & Wagner, 2007; Trambaiolli et al., 2022; Wright et al., 2008; Zhang et al., 2022), it is possible that targeted stimulation to this region may have partially compensated for these abilities typically afforded by higher IQ. Future studies with larger samples and individual difference measures are needed to adjudicate between these interpretations.

#### Awareness and fact integration

In addition to the unique stimulation effects, this study revealed a significant facilitation effect related to participants’ awareness of fact pairs. Specifically, for both stimulation groups, integration scores improved once participants became more aware of the associations between fact pairs. This aspect was not addressed in previous studies using the self-derivation paradigm. While few studies on associative learning have measured participants’ awareness of fact pairs, it remains an important cognitive component to consider when designing such experiments. Previous studies have shown that awareness could influence participants’ performance in semantic processing. For instance, a previous study by Kumaran & Ludwig (2013) showed that participants’ awareness of task hierarchy, rather than relation between pairs, predicted higher learning accuracy. Their results contrast with our finding that awareness towards fact pairs enhanced integration performance. This discrepancy suggests that different experimental designs may produce varying effects of awareness. Additionally, differences in the sets of questions used to assess awareness between studies could also account for the varying results.

### Limitations

Our study has several limitations. First, although our sample size in Experiment 1 was informed by previous HD-tDCS stimulation study using semantic learning paradigm (*e.g*., Price et al., 2016), and the sample size for Experiment 2 was preregistered based on *a priori* power analysis, the relatively modest samples may still constrain the generalizability of the findings and the ability to detect smaller effects. Because Experiment 1 was exploratory, determining the exact sample size required to achieve significant effects was challenging. Nevertheless, we used cortical electric field models in SimNIBS to optimize stimulation targeting (Thielscher, Antunes & Saturnino, 2015), which increases confidence in the focality of our approach. Finally, although our hypotheses regarding secondary factors (such as IQ and awareness) were pre-specified and preregistered in Experiment 2, detecting individual difference effects typically requires substantially larger statistical power and formal moderation analysis. Future studies with larger independent samples would be critical to establish the robustness of the observed relationships involving IQ and awareness.

Second, our investigation focused exclusively on the left AG and left vlPFC, as these regions were hypothesized to play causal roles in semantic integration and inference. While the observed differential effects highlight their functional contributions, the absence of concurrent neuroimaging limits our ability to clarify the underlying neural mechanisms. For example, it remains unclear why participants with lower baseline performance benefited more from anodal stimulation to the left AG, or whether such effects extend to the broader semantic network. Future research should combine non-invasive stimulation with neuroimaging methods to capture neural activity changes and provide a more mechanistic account of these effects.

## Conclusions

In conclusion, the present study utilized anodal HD-tDCS to investigate the roles of the left AG and left vlPFC in semantic integration. Although stimulation to these regions did not significantly alter mean self-derivation performance, the results revealed distinct patterns of modulation for each site. In an exploratory observation, stimulation of the left AG was associated with a significant reduction in performance variance, appearing to raise the performance floor for lower-performing participants. In contrast, testing our pre-registered hypothesis in Experiment 2 showed that stimulation of the left vlPFC attenuated the typical relationship between estimated IQ and integration scores. While these findings do not support a direct causal enhancement of semantic integration by these regions, they suggest that the AG and vlPFC may differentially modulate how individual cognitive resources are deployed during self-derivation. Future research with concurrent neuroimaging is needed to further clarify the precise neural dynamics and network-level interactions underlying these modulatory effects.

## Supplemental Information

10.7717/peerj.21761/supp-1 Supplemental Information 1 Questionnaires.

## Additional Information and Declarations

### Data Availability

The following information was supplied regarding data availability:

The data is available at Open Science Framework (OSF): Zhang, X., Joseph, K., Gilboa, A., & Lochner, M. (2025, November 4). Data. https://doi.org/10.17605/OSF.IO/NV4T3.

## References

[ref-1] Alba JW, Hasher L (1983). Is memory schematic?. Psychological Bulletin.

[ref-2] Azarpaikan A, Taherii Torbati H, Sohrabi M, Boostani R, Ghoshuni M (2019). Power spectral parameter variations after transcranial direct current stimulation in a bimanual coordination task. Adaptive Behavior.

[ref-3] Badre D, Poldrack RA, Paré-Blagoev EJ, Insler RZ, Wagner AD (2005). Dissociable controlled retrieval and generalized selection mechanisms in ventrolateral prefrontal cortex. Neuron.

[ref-4] Badre D, Wagner AD (2007). Left ventrolateral prefrontal cortex and the cognitive control of memory. Neuropsychologia.

[ref-5] Bauer P (2020). Bauer lab integration and self-derivation stimulus (BLISS) bank.

[ref-6] Bauer PJ, Blue SN, Xu A, Esposito AG (2016). Productive extension of semantic memory in school-aged children: relations with reading comprehension and deployment of cognitive resources. Developmental Psychology.

[ref-7] Bauer PJ, Esposito AG, Daly JJ (2020). Self-derivation through memory integration: a model for accumulation of semantic knowledge. Learning and Instruction.

[ref-8] Bauer PJ, Varga NL (2017). Similarity and deviation in event segmentation and memory integration: commentary on Richmond, Gold, & Zacks. Journal of Applied Research in Memory and Cognition.

[ref-9] Becker M, Cabeza R (2025). The neural basis of the insight memory advantage. Trends in Cognitive Sciences.

[ref-10] Bein O, Reggev N, Maril A (2020). Prior knowledge promotes hippocampal separation but cortical assimilation in the left inferior frontal gyrus. Nature Communications.

[ref-11] Benedek M, Kenett YN, Umdasch K, Anaki D, Faust M, Neubauer AC (2017). How semantic memory structure and intelligence contribute to creative thought: a network science approach. Thinking & Reasoning.

[ref-12] Binder JR, Desai RH (2011). The neurobiology of semantic memory. Trends in Cognitive Sciences.

[ref-13] Binder JR, Desai RH, Graves WW, Conant LL (2009). Where is the semantic system? A critical review and meta-analysis of 120 functional neuroimaging studies. Cerebral Cortex.

[ref-14] Binkofski FC, Klann J, Caspers S (2016). On the neuroanatomy and functional role of the inferior parietal lobule and intraparietal sulcus. Neurobiology of Language.

[ref-15] Bonner MF, Peelle JE, Cook PA, Grossman M (2013). Heteromodal conceptual processing in the angular gyrus. NeuroImage.

[ref-16] Bonnici HM, Richter FR, Yazar Y, Simons JS (2016). Multimodal feature integration in the angular gyrus during episodic and semantic retrieval. The Journal of Neuroscience: The Official Journal of the Society for Neuroscience.

[ref-17] Branzi FM, Lambon Ralph MA (2023). Semantic-specific and domain-general mechanisms for integration and update of contextual information. Human Brain Mapping.

[ref-18] Branzi FM, Pobric G, Jung J, Lambon Ralph MA (2021). The left angular gyrus is causally involved in context-dependent integration and associative encoding during narrative reading. Journal of Cognitive Neuroscience.

[ref-19] Burnham KP, Anderson DR (2004). Multimodel inference: understanding AIC and BIC in model selection. Sociological Methods & Research.

[ref-20] Caspers S, Eickhoff SB, Geyer S, Scheperjans F, Mohlberg H, Zilles K, Amunts K (2008). The human inferior parietal lobule in stereotaxic space. Brain Structure and Function.

[ref-21] Chen Y, Wang C, Song P, Sun C, Zhang Y, Zhao X, Du J (2022). Alpha rhythm of electroencephalography was modulated differently by three transcranial direct current stimulation protocols in patients with ischemic stroke. Frontiers in Human Neuroscience.

[ref-22] Cognition (n.d). Run experiments online. https://www.cognition.run/.

[ref-23] Collins DL, Neelin P, Peters TM, Evans AC (1994). Automatic 3D intersubject registration of MR volumetric data in standardized Talairach space. Journal of Computer Assisted Tomography.

[ref-24] Craik FI, Bialystok E (2006). Cognition through the lifespan: mechanisms of change. Trends in Cognitive Sciences.

[ref-25] Dedoncker J, Brunoni AR, Baeken C, Vanderhasselt MA (2016). A systematic review and meta-analysis of the effects of transcranial direct current stimulation (tDCS) over the dorsolateral prefrontal cortex in healthy and neuropsychiatric samples: influence of stimulation parameters. Brain Stimulation.

[ref-26] Esposito AG, Bauer PJ (2017). Going beyond the lesson: self-generating new factual knowledge in the classroom. Journal of Experimental Child Psychology.

[ref-27] Esposito AG, Bauer PJ (2019). Self-derivation through memory integration under low surface similarity conditions: the case of multiple languages. Journal of Experimental Child Psychology.

[ref-28] Faul F, Erdfelder E, Buchner A, Lang AG (2009). Statistical power analyses using G*Power 3.1: tests for correlation and regression analyses. Behavior Research Methods.

[ref-29] Fernández G, Morris RGM (2018). Memory, novelty and prior knowledge. Trends in Neurosciences.

[ref-30] Gilboa A, Marlatte H (2017). Neurobiology of schemas and schema-mediated memory. Trends in Cognitive Sciences.

[ref-31] Gilboa A, Sekeres M, Moscovitch M, Winocur G (2014). Higher-order conditioning is impaired by hippocampal lesions. Current Biology.

[ref-32] Gilboa A, Sekeres M, Moscovitch M, Winocur G (2019). The hippocampus is critical for value-based decisions guided by dissociative inference. Hippocampus.

[ref-33] Guo D, Yang J (2023). Reactivation of schema representation in lateral occipital cortex supports successful memory encoding. Cerebral Cortex.

[ref-34] Hebscher M, Wing E, Ryan J, Gilboa A (2019). Rapid cortical plasticity supports long-term memory formation. Trends in Cognitive Sciences.

[ref-35] Heckers S, Zalesak M, Weiss AP, Ditman T, Titone D (2004). Hippocampal activation during transitive inference in humans. Hippocampus.

[ref-36] Hintzman DL (1986). “Schema abstraction” in a multiple-trace memory model. Psychological Review.

[ref-37] Hoffman P, Jefferies E, Lambon Ralph MA (2010). Ventrolateral prefrontal cortex plays an executive regulation role in comprehension of abstract words: convergent neuropsychological and repetitive TMS evidence. Journal of Neuroscience.

[ref-38] Humphreys GF, Lambon Ralph MA, Simons JS (2021). A unifying account of angular gyrus contributions to episodic and semantic cognition. Trends in Neurosciences.

[ref-39] Jensen AR (2006). Clocking the mind: mental chronometry and individual differences.

[ref-40] Kovacs K, Conway ARA (2016). Process overlap theory: a unified account of the general factor of intelligence. Psychological Inquiry.

[ref-41] Kuhnke P, Chapman CA, Cheung VKM, Turker S, Graessner A, Martin S, Williams KA, Hartwigsen G (2022). The role of the angular gyrus in semantic cognition: a synthesis of five functional neuroimaging studies. Brain Structure and Function.

[ref-42] Kumaran D, Ludwig H (2013). Transitivity performance, relational hierarchy knowledge and awareness: results of an instructional framing manipulation. Hippocampus.

[ref-43] Kuo HI, Bikson M, Datta A, Minhas P, Paulus W, Kuo MF, Nitsche MA (2013). Comparing cortical plasticity induced by conventional and high-definition 4 × 1 ring tDCS: a neurophysiological study. Brain Stimulation.

[ref-44] Lambon Ralph MA, Jefferies E, Patterson K, Rogers TT (2016). The neural and computational bases of semantic cognition. Nature Reviews Neuroscience.

[ref-45] Lee K, Ng SF, Bull R, Pe ML, Ho RHM (2011). Are patterns important? An investigation of the relationships between proficiencies in patterns, computation, executive functioning, and algebraic word problems. Journal of Educational Psychology.

[ref-46] Levy DA (2012). Towards an understanding of parietal mnemonic processes: some conceptual guideposts. Frontiers in Integrative Neuroscience.

[ref-47] Ling MKD, Loh SC (2021). Relationships between cognitive pattern recognition and specific mathematical domains in mathematics education. International Journal of Mathematical Education in Science and Technology.

[ref-48] Luchini SA, Wang S, Kenett YN, Beaty RE (2024). Mapping the memory structure of high-knowledge students: a longitudinal semantic network analysis. Journal of Intelligence.

[ref-49] Mackintosh N (2011). IQ and human intelligence.

[ref-50] Marlatte H, Belchev Z, Fraser M, Gilboa A (2024). The effect of hippocampal subfield damage on rapid temporal integration through statistical learning and associative inference. Neuropsychologia.

[ref-51] Meltzer JA, Sivaratnam G, Deschamps T, Zadeh M, Li C, Farzan F, Francois-Nienaber A (2024). Contrasting MEG effects of anodal and cathodal high-definition TDCS on sensorimotor activity during voluntary finger movements. Frontiers in Neuroimaging.

[ref-52] Menenti L, Petersson KM, Scheeringa R, Hagoort P (2009). When elephants fly: differential sensitivity of right and left inferior frontal gyri to discourse and world knowledge. Journal of Cognitive Neuroscience.

[ref-53] Merhav M, Karni A, Gilboa A (2015). Not all declarative memories are created equal: fast mapping as a direct route to cortical declarative representations. NeuroImage.

[ref-54] Miller-Goldwater HE, Cronin-Golomb LM, Porter BM, Bauer PJ (2021). Developmental differences in reactivation underlying self-derivation of new knowledge through memory integration. Cognitive Psychology.

[ref-55] Molinaro N, Paz-Alonso PM, Duñabeitia JA, Carreiras M (2015). Combinatorial semantics strengthens angular-anterior temporal coupling. Cortex: A Journal Devoted to the Study of the Nervous System and Behavior.

[ref-56] Neurosynth (n.d). Neurosynth.org. https://neurosynth.org/.

[ref-57] Nitsche MA, Paulus W (2000). Excitability changes induced in the human motor cortex by weak transcranial direct current stimulation. The Journal of Physiology.

[ref-58] Piaget J (1952). Introduction: the biological problem of intelligence. The Origins of Intelligence in Children.

[ref-59] Preston AR, Eichenbaum H (2013). Interplay of hippocampus and prefrontal cortex in memory. Current Biology.

[ref-60] Price AR, Bonner MF, Peelle JE, Grossman M (2015). Converging evidence for the neuroanatomic basis of combinatorial semantics in the angular gyrus. The Journal of Neuroscience: The Official Journal of the Society for Neuroscience.

[ref-61] Price AR, Peelle JE, Bonner MF, Grossman M, Hamilton RH (2016). Causal evidence for a mechanism of semantic integration in the angular gyrus as revealed by high-definition transcranial direct current stimulation. The Journal of Neuroscience.

[ref-62] R Core Team (2019). R: a language and environment for statistical computing.

[ref-63] Saturnino GB, Siebner HR, Thielscher A, Madsen KH (2019). Accessibility of cortical regions to focal TES: dependence on spatial position, safety, and practical constraints. NeuroImage.

[ref-64] Schapiro AC, Turk-Browne NB, Botvinick MM, Norman KA (2017). Complementary learning systems within the hippocampus: a neural network modelling approach to reconciling episodic memory with statistical learning. Philosophical Transactions of the Royal Society of London. Series B, Biological Sciences.

[ref-65] Schlichting ML, Preston AR (2015). Memory integration: neural mechanisms and implications for behavior. Current Opinion in Behavioral Sciences.

[ref-66] Schlichting ML, Zeithamova D, Preston AR (2014). CA1 subfield contributions to memory integration and inference. Hippocampus.

[ref-67] Seghier ML (2012). The angular gyrus. The Neuroscientist.

[ref-68] Shelton JT, Elliott EM, Matthews RA, Hill BD, Gouvier WD (2010). The relationships of working memory, secondary memory, and general fluid intelligence: working memory is special. Journal of Experimental Psychology. Learning, Memory, and Cognition.

[ref-69] Sommer T (2017). The emergence of knowledge and how it supports the memory for novel related information. Cerebral Cortex.

[ref-70] Spitoni GF, Cimmino RL, Bozzacchi C, Pizzamiglio L, Di Russo F (2013). Modulation of spontaneous alpha brain rhythms using low-intensity transcranial direct-current stimulation. Frontiers in Human Neuroscience.

[ref-71] Thielscher A, Antunes A, Saturnino GB (2015). Field modeling for transcranial magnetic stimulation: a useful tool to understand the physiological effects of TMS?.

[ref-72] Thorndyke PW, Yekovich FR (1980). A critique of schema-based theories of human story memory. Poetics.

[ref-73] Trambaiolli LR, Peng X, Lehman JF, Linn G, Russ BE, Schroeder CE, Liu H, Haber SN (2022). Anatomical and functional connectivity support the existence of a salience network node within the caudal ventrolateral prefrontal cortex. ELife.

[ref-74] Tse D, Langston RF, Kakeyama M, Bethus I, Spooner PA, Wood ER, Witter MP, Morris RGM (2007). Schemas and memory consolidation. Science.

[ref-75] van der Linden M, Berkers RMWJ, Morris RGM, Fernández G (2017). Angular gyrus involvement at encoding and retrieval is associated with durable but less specific memories. The Journal of Neuroscience.

[ref-76] van Kesteren MTR, Ruiter DJ, Fernández G, Henson RN (2012). How schema and novelty augment memory formation. Trends in Neurosciences.

[ref-77] Varga NL, Cronin-Golomb L, Bauer PJ (2022). Self-derivation of new knowledge through memory integration varies as a function of prior knowledge. Memory.

[ref-78] Varga NL, Esposito AG, Bauer PJ (2019). Cognitive correlates of memory integration across development: explaining variability in an educationally relevant phenomenon. Journal of Experimental Psychology: General.

[ref-79] Varga NL, Manns JR (2021). Delta-modulated cortical alpha oscillations support new knowledge generation through memory integration. NeuroImage.

[ref-80] Wang SH, Tse D, Morris RGM (2012). Anterior cingulate cortex in schema assimilation and expression. Learning & Memory.

[ref-81] Webb WG, Webb WG (2017). Central language mechanism and learning. Neurology for the Speech-Language Pathologist.

[ref-82] Wechsler D (2011). Wechsler abbreviated scale of intelligence (WASI-II).

[ref-83] Wilson JT, Bauer PJ (2021). Prompt-facilitated learning: the development of unprompted memory integration and subsequent self-derivation. Memory & Cognition.

[ref-84] Wing EA, Burles F, Ryan JD, Gilboa A (2022). The structure of prior knowledge enhances memory in experts by reducing interference. Proceedings of the National Academy of Sciences of the United States of America.

[ref-85] Wing EA, Gilboa A, Ryan JD (2024). Interactive influences of prior knowledge on episodic memory. iScience.

[ref-87] Wright SB, Matlen BJ, Baym CL, Ferrer E, Bunge SA (2008). Neural correlates of fluid reasoning in children and adults. Frontiers in Human Neuroscience.

[ref-88] Yarkoni T, Poldrack RA, Nichols TE, Van Essen DC, Wager TD (2011). Large-scale automated synthesis of human functional neuroimaging data. Nature Methods.

[ref-89] Zeithamova D, Schlichting ML, Preston AR (2012). The hippocampus and inferential reasoning: building memories to navigate future decisions. Frontiers in Human Neuroscience.

[ref-90] Zhang X, Lochner M, Joseph K, Gilboa A (2023). Neural substrates of knowledge integration: insights from AG and vlPFC stimulation. Open Science Framework.

[ref-91] Zhang M, Nathaniel U, Savill N, Smallwood J, Jefferies E (2022). Intrinsic connectivity of left ventrolateral prefrontal cortex predicts individual differences in controlled semantic retrieval. NeuroImage.

