# Supplementary material for: Neural substrates of knowledge integration: differential effects of high-definition transcranial direct current stimulation (HD-tDCS) on angular gyrus and ventrolateral prefrontal cortex
Source: PeerJ. 2026 Oct 6;14:e21761. doi: 10.7717/peerj.21761 (PMC13651779; doi:10.7717/peerj.21761)
Supplement: Supplementary file 1 [file peerj-14-21761-s001.pdf]

Supplementary Materials

Post-experiment questionnaire: awareness assessment

Experiment 1

Questionnaire On Side Effects For Brain Stimulation Studies

Study number: __________ Participant ID: _________ Date: __________

1. In order to track the possible occurrence of side effects in brain stimulation experiments, we ask you to report to us if you experienced any of the following adverse effects during or after your participation in the experiment.

**Side effects**	**Check Box**
Headache	
Facial, dental, or eye pain	
Neck pain / stiffness	
Hearing problem / ear pain	
Skin irritation	
Pain / stiffness elsewhere	
Seizure	
Syncope / fainting / lightheadedness	
Nausea / vertigo / dizziness	
Mood change (depression, euphoria)	
Other (please describe below)	

If you experienced **no adverse effects**, please check this box: ☐
If you answered "yes" to any of the above, please provide details:

____

____

2. Half of our participants receive real stimulation and half receive sham stimulation, i.e. minimal stimulation that should not have a significant physiological effect, to control for possible placebo effects. We cannot tell you which group you were in, but would like to know:

During stimulation, did you think you were getting real stimulation? Yes / No

Please estimate the likelihood that you were in one of these groups:

Definitely sham stimulation	Probably sham	Maybe sham	I have no idea	Maybe real	Probably real	Definitely real stimulation
3	2	1	0	1	2	3

3. Did you notice anything odd or interesting about the facts that your learned?

4. What strategies did you use to answer the questions during the knowledge assessment?

Experiment 2

Questionnaire On Side Effects For Brain Stimulation Studies

Study number: __________ Participant ID: _________ Date: __________

1. In order to track the possible occurrence of side effects in brain stimulation experiments, we ask you to report to us if you experienced any of the following adverse effects during or after your participation in the experiment.

Side effects	Check Box
Headache	
Facial, dental, or eye pain	
Neck pain / stiffness	
Hearing problem / ear pain	
Skin irritation	
Pain / stiffness elsewhere	
Seizure	
Syncope / fainting / lightheadedness	
Nausea / vertigo / dizziness	
Mood change (depression, euphoria)	
Other (please describe below)	

If you experienced **no adverse effects**, please check this box: ☐
If you answered "yes" to any of the above, please provide details:
____
____

2. Half of our participants receive real stimulation and half receive sham stimulation, i.e. minimal stimulation that should not have a significant physiological effect, to control for possible placebo effects. We cannot tell you which group you were in, but would like to know:

During stimulation, did you think you were getting real stimulation? Yes / No

Please estimate the likelihood that you were in one of these groups:

Definitely sham stimulation	Probably sham	Maybe sham	I have no idea	Maybe real	Probably real	Definitely real stimulation
3	2	1	0	1	2	3

3. Did you notice anything odd or interesting about the facts that your learned?

4. What strategies did you use to answer the questions during the knowledge assessment?

Think back to the **first phase** of the experiment, when the facts were presented one by one:

5. As you were reading the facts during the first study phase, did you notice any relationship between them?

6. On a scale of 1-5, as you were reading, how related did you think some of these facts were? (1 - not related at all, 5 - very related)

1 2 3 4 5

7. If you thought some of the facts were related as you were reading them, what did you think that relation was? Why did you think the related facts were presented? (Note: leave blank if you did not think about the relationship between the facts during the initial presentation.)

8. On a scale of 1-5, how focused were you when learning these facts? (1 - not focused at all, 5 - very focused)

1 2 3 4 5

9. Explain what factors may have made you focused or unfocused on the task (e.g., I was not super focused because I am sleep-deprived / I was very focused because facts are interesting to me, and etc.).

Awareness scoring

For Experiment 1, participants' answers for question 3 & 4 were collected from this survey and scored dichotomously. If participants mentioned anything related to "some facts were related", or "there was an association between some facts", or "I tried to associate some pairs of facts during the test", it was considered that participants were aware of the fact pair associations (i.e., a score of 1). Otherwise, participants received a total awareness score of 0.

A few additional questions were added to Experiment 2 post-experiment questionnaire. To ensure that participants understand the questions more explicitly, question 5 to 7 were added to more directly prompt for participants awareness. These questions are shown on the second page of the questionnaire so that participants always answered implicit awareness question before answering the explicit ones. Based on questions 3, 4, 5, and 7, if participants mentioned anything related to "I notice some facts were related" or "I tried to associate fact pairs for the task", they received a score of 1. Otherwise, they received a score of 0. Question 8 and 9 were added to assess participants' attentiveness to the task.

Familiarity control

While participants were performing the encoding task, their responses to each question were recorded. The question was shown after the fact, and participants were prompted with "Is this a familiar fact?" or "Did you know this fact before?". Participants' responses to these questions were recorded as dichotomous scores (e.g., 1 for yes, and 0 for no). Familiarity to facts were summed across each participant (e.g., for a total of 60). A percentage was then calculated by the total number of facts they mentioned that they knew divided by 60.

Blinding effectiveness check

For the post-stimulation questionnaire, participants were asked whether they noticed if they were getting real stimulation (i.e., question 2). Their responses on the Likert scale were collected and analyzed independent sample t test in R (version 3.6.0, R Core Team, 2019).

Exploratory non-parametric analysis

To descriptively explore the distribution of integration scores across different cognitive profiles, we performed an exploratory median-split visualization. These descriptive comparisons are provided to allow for qualitative comparison with prior literature (e.g., Esposito & Bauer, 2017; Varga et al., 2019; Varga & Bauer, 2021) which suggests that baseline performance levels may qualitatively influence neurostimulation outcomes.

To understand this reduced correlation between IQ and integration scores, we conducted an exploratory non-parametric test as follows. We split participants within each group into low- and high-IQ subgroups using each group's median IQ scores. We then conducted a non-parametric Aligned Rank Transform test using the ARTool package (Wobbrock et al., 2011). The aligned rank transformed data was subjected to ANOVA with 10,000 resample permutations which revealed no effect of stimulation ($F(1, 47) = 0.29$; $p_{permutation} = 0.591$), a significant effect of median IQ split ($F(1, 47) = 7.14$; $p_{permutation} = .010$) and a trending group by median IQ split interaction ($F(1, 47) = 3.63$; $p_{permutation} = .063$) (Fig. 5C). Given this trend, we conducted pairwise permutation tests to characterize its source. These tests showed a significant difference in integration scores between low- and high-IQ participants within the sham group (permutation statistic = 3.49; $p_{permutation} < .001$), while there was no difference between high and low IQ participants within the stimulation group (permutation statistic = 0.41; $p_{permutation} = .707$). Importantly, these pairwise results do not indicate a statistically significant interaction, but rather suggest that the observed group difference in IQ-performance correlations may be driven by the result of stimulation to vlPFC, making the integration performance of individuals with low IQ more similar to that of those with high IQ.
